# The Haze Nightmare Following the Economic Boom in China: Dilemma and Tradeoffs

**DOI:** 10.3390/ijerph13040402

**Published:** 2016-04-02

**Authors:** Jian Sun, Jinniu Wang, Yanqiang Wei, Yurui Li, Miao Liu

**Affiliations:** 1 Institute of Geographic Sciences and Natural Resources Research, Chinese Academy of Sciences, Beijing 100101, China; sunjian@igsnrr.ac.cn (J.S.); liyr@igsnrr.ac.cn (Y.L.); 2 Chengdu Institute of Biology, Chinese Academy of Sciences, Chengdu 610041, China; wangjn@cib.ac.cn; 3 Key Laboratory of Mountain Ecological Restoration and Bioresource Utilization, Chinese Academy of Sciences, Chengdu 610041, China; 4 Ecological Restoration Biodiversity Conservation Key Laboratory of Sichuan Province, Chengdu 610041, China; 5 International Centre for Integrated Mountain Development (ICIMOD), G.P.O. Box 3226, Kathmandu, Nepal; 6 Cold and Arid Regions Environmental and Engineering Research Institute, Chinese Academy of Sciences, Lanzhou 730000, China; 7 State Key Laboratory of Urban and Regional Ecology, Research Center for Eco-Environmental Sciences, Chinese Academy of Sciences, Beijing 100085, China; miaoliu@rcees.ac.cn

**Keywords:** haze, industrial soot emissions, SO_2_ emissions, PM_2.5_, pertussis and pulmonary tuberculosis, policy framework

## Abstract

This study aims to expand on a deeper understanding of the relationship between rapid economic development and ensuing air pollution in China. The database includes the gross domestic product (GDP), the value added of a secondary industry, the *per capita* GDP (PGDP), greenhouse gases emissions, and PM_2.5_ concentrations. The results indicate that China’s PGDP has continued to rise over the past decade, and the rate of PGDP slowed down from 1980 to 2004 (slope = 5672.81, *R*^2^ = 0.99, *p* < 0.001) but was significantly lower than that from the year 2004 to 2013 (slope = 46,911.08, *R*^2^ > 0.99, *p* < 0.001). Unfortunately, we found that total coal consumption, annual steel production, and SO_2_ emission had been continually growing as the overall economy expands at temporal scale, with the coefficient of determinations greater than 0.98 (*p* < 0.001). Considering the spatial pattern aspect, we also found a significant relationship between GDP and greenhouse gases. Meanwhile, severe air pollution has negatively impacted the environment and human health, particularly in some highlighted regions. The variation explained by both total SO_2_ emission and total smoke and dust emission were 33% (*p* < 0.001) and 24% (*p* < 0.01) for the rate of total pertussis at temporal scale, respectively. Furthermore, at the spatial scale, pulmonary tuberculosis rates and pertussis mainly occurred in area with serious air pollution (economically developed region). It can be summarized that the extensive mode of economic growth has brought a number of serious environment and human health problems. Thus, a new policy framework has been proposed to meet the goals of maintaining a healthy economy without harming natural environment, which may prove integral, especially when coupled with long-term national strategic development plans.

## 1. Introduction

China is the most populated and the fastest developing major country in the history of the world. Since the 1980s, primary energy consumption and carbon dioxide emissions have demonstrated a drastic growth trend in China [1]. Currently, the rapid economy growth of China is said to have massive implications on the energy demand and associated environmental issues [2]. In 2008, China accounted for over 17.2% of the global total energy consumption, making China the second largest energy consumer in the world, after the United States. The majority of China’s consumption consists of fossil fuels, with renewable energy taking up less than 8%. The combustion of fossil fuels has generated considerable greenhouse gas (GHG) and particulate matter (e.g., black carbon) emissions, which not only contributes to global warming but is the major source of haze. It has been well known for its deleterious health impacts and as an obstacle to sustainable development.

In China, air pollution has recently become quite severe; aerosol loading in China has increased considerably due to the increasing population, urbanization, and industrialization. Atmospheric aerosols, particularly small particles with diameters between 0.1 and 1 mm, interact strongly with short wavelengths of light, leading to low-visibility conditions, identified as haze at high atmospheric concentrations [3], and giving rise to serious environmental problems on both regional and global scales. For instance, the appearance of haze can be seen in Guangzhou approximately 278 days per year on average over a 4-year period (2008–2011) [4]. In January 2013, a severe fog and haze event of great intensity, long duration, and extensive coverage occurred in eastern China [5]. A NASA (The National Aeronautics and Space Administration) satellite map highlights the severity of the problem: from a global perspective, eastern China suffered the highest average PM_2.5_ concentrations in the air from 2001 to 2006 [6]. Meanwhile, haze pollution has significant health impact and was associated with an increase in an excessive risk of hospital admissions [4]. Thus, more attention should be paid to the health effects of haze.

Poverty reduction and environmental protection are global tasks crucial for sustainable development [7]. However, China has maintained rapid economic growth to boost incomes and employ a rapidly urbanizing population as the top priority in the country in recent decades. As a result, economic growth in China is heavily dependent on infrastructure investment and development of energy-intensive industries. It is thus critical to promote technological progress and adjust the energy structure to improve energy efficiency and reduce energy consumption [1]. Unfortunately, haze and fog in China have hit record levels; the country has been suffering from the worst air pollution since 1961 [8]. In particular, low energy efficiency, energy shortages, and energy-related environmental issues are becoming critical constraints to sustainable development in China. It is essential to develop global policies under climate change background and insight from other emerging economies with low pollutant emission in future, which will be beneficial to understand key drivers of growing energy consumption coupled with carbon emissions in China.

This study aims to address several inherent mechanisms and interactions between booming economic development and energy consumption as well as spatial-temporal air pollution in China, specifically seeking to accomplish goals as follows: (1) to analyze the spatial-temporal distribution of economic development by conducting gross domestic product (GDP) and energy consumption, as well as their correlation in time series and region-specifically across China; (2) to explore regional air pollution and haze patterns in China, and their relationship with respiratory diseases and economic development; and (3) to outline policy suggestions containing relevant laws, regulations, and guidelines.

## 2. Materials and Methods

### 2.1. Data Collection

All of the data (GDP, total coal and petroleum consumption, total SO_2_ emissions, and total industrial soot emissions of the cities) were obtained from the National Bureau of Statistics in China [9]. The GDP, value added of secondary industry (VASI), and *per capita* GDP (PGDP) were collected from 1980 to 2013. In addition, total SO_2_ emissions were compiled from trends observed between 1980 and 2013, and data pertaining to industrial soot emissions and PM_2.5_ (*China City Statistical Yearbook 2013*) in different cities were obtained from documents in 2012 and 2013, respectively. Here, the industrial soot emissions only refer to the anthropogenic soot emissions of industry rather than the natural small solid particles, e.g., sand storm particles. The information on annual density of SO_2_, NO_2_, CO, and PM_2.5_ are extracted from the *China Statistical Yearbook on Environment* 2014 [10]. Meanwhile, total coal consumption and China’s demand for steel from 1980 to 2014, were obtained from the National Bureau of Statistics in China [9].

### 2.2. Tools of Analysis

In this study, ArcGIS 10.2 (ESRI, Inc., Redlands, CA, USA) was used to draw spatial graphs and SigmaPlot for Windows version 10.0 (Systat Software, Inc., Chicago, IL, USA) to conduct correlation and regression analysis. Correlations between different variables were determined using two-tailed Pearson’s Correlation at *p* = 0.05 significance.

## 3. Results

### 3.1. Temporal Dynamics of Air Pollution, National Economic and Social Development

**Figure 1:**
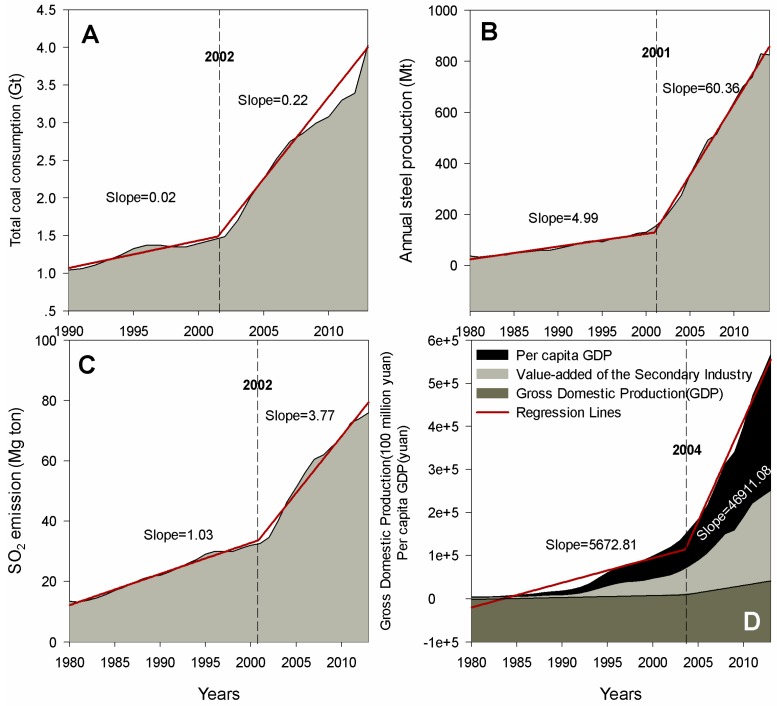
Annual coal consumption (1990–2014 (**A**)); the demand for steel (1980–2014 (**B**)); the total SO_2_ emission (1980–2013 (**C**)); and the economic growth (1980–2013 (**D**)). All documents are extracted from the *China Statistical Yearbook* (1980–2014) [9].

In 2014 coal consumption in China officially fell by 2.9% for the first time in the past 14 years (Figure 1A), and the year 2002 can be regarded as a “tipping point”, with slopes of 0.02 Gt/year (*Y* = 0.02*X* − 37.47) and 0.22 Gt/year (*Y* = 0.22*X* − 434.83) before and after 2002 (Table 1, *R*^2^ = 0.99, *p* < 0.001), respectively. Meanwhile, in light of the significant positive trends of steel production in time series (Figure 1B), the year 2001 can be regarded as a “tipping point”, with slopes of 4.99 Mt/year (*Y* = 4.99*X* − 9849.96) and 60.36 Mt/year (*Y* = 60.36*X* − 120,650.98) before and after 2001 (Table 1, *R*^2^ = 0.99, *p* < 0.001), respectively. From 1980 to 2013 (Figure 1C), total sulfur dioxide (SO_2_) emissions increased continuously, nearly exceeding a four-fold increase and even more at the national level. Similarly, total SO_2_ emissions demonstrated sharply increasing trends after the year 2001; moreover, the rate of total SO_2_ emissions significantly boosted from the year 2000 to 2013 (*Y* = 3.77*X* + 5405.29, Table 1, *R*^2^ = 0.99, *p* < 0.001), while had been slower process from 1980 to 2000 (*Y* = 1.03*X* − 2022.61) Table 1. The economic growth in China was significantly slower from 1980 to 2004 than that from 2004 to 2013. GDP, VASI, and PGDP all demonstrated sharply increasing trends after the year 2004 (Figure 1D). In addition, the maximum GDP, VASI, and PGDP amounted to 568.85 billion Yuan, 41.91 billion Yuan and 249.68 Yuan, respectively. The GDP, VASI, and PGDP of China have been continuously expanding over the past decade. It can be referred segmentally that the PGDP has increased to 41,907.51 Yuan per year, prior to 2004, at which point they boomed up to 12,335.58 Yuan per year (Table 1).

**Figure 2:**
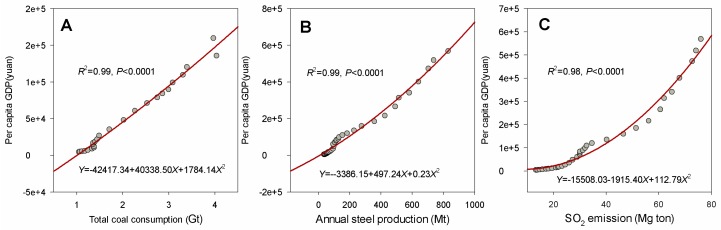
The quadratic regression analysis of the total coal consumption (**A**); the annual steel production (**B**); the SO_2_ emission (**C**) with the per capita GDP. All documents are extracted from the *China Statistical Yearbook* (1980–2014) [9].

As shown in Figure 2, there exists a close relationship of PGDP with total coal consumption (Figure 2A), annual steel production (Figure 2B), and SO_2_ emission (Figure 2C). The fitting functions were *Y* = 42,417.34 + 40,338.50*X* + 1784.14*X*^2^ (*R*^2^ = 0.99, *p* < 0.001), *Y* = 3386.15 + 497.24*X* + 0.23*X*^2^ (*R*^2^ = 0.99, *p* < 0.001), and *Y* = 15,508.03 + 1915.40*X* + 112.79*X*^2^ (*R*^2^ = 0.98, *p* < 0.001), respectively. We found that total coal consumption, annual steel production, and SO_2_ emission had been continually growing along with the growing overall economy.

### 3.2. Spatial Dynamics of Air Pollution, National Economic and Social Development

**Figure 3:**
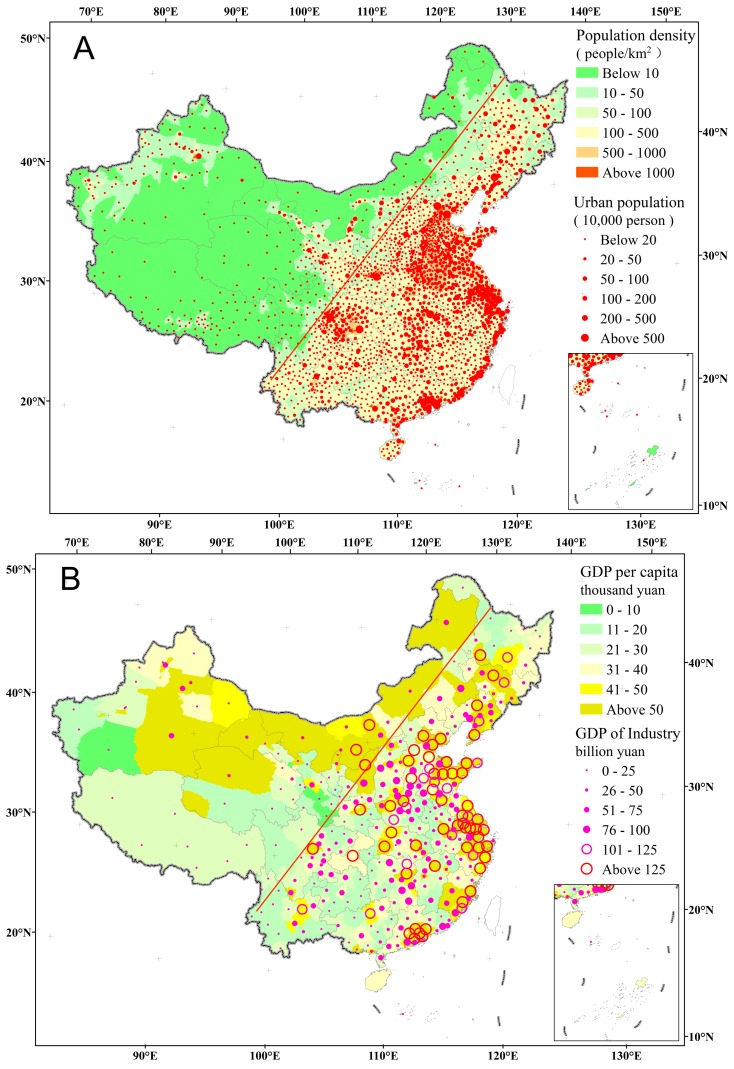
The spatial pattern of population density (**A**); and the spatial pattern of GDP per capita and GDP of industry (**B**). All documents are extracted from the *China Statistical Year Book for Regional Economy* (2014) [9].

Based on the national census in 2013, more than 94% of the population is distributed throughout the southwest (below the Hu Huanyong line, Figure 3A); the densest population and urban population are distributed mainly across the Bohai coastal region, the Yangtze River delta, the Pearl River delta, the eastern coastal and the plains area. In terms of per capita GDP and industrial added value, high value areas are also mainly distributed in the eastern coastal and inland super-large cities (Figure 3B).

**Figure 4:**
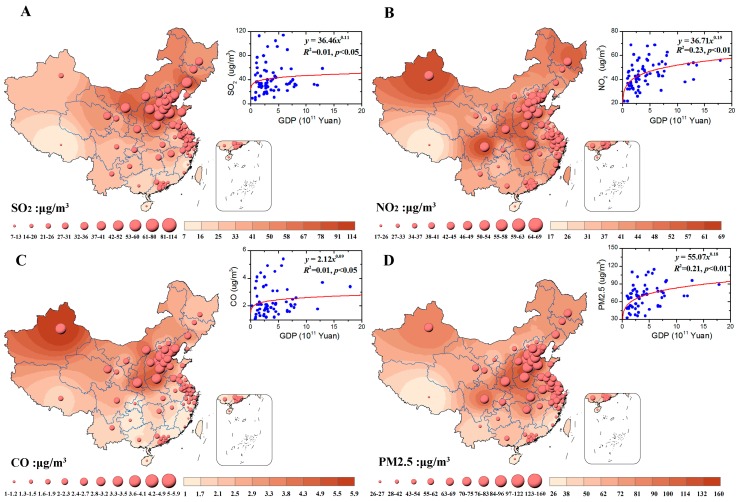
The spatial distributions of the annual density of the SO_2_ (**A**); NO_2_ (**B**); CO (**C**); and PM_2.5_ (**D**) in 2013. The little panels present the relationships of GDP with SO_2_ (**A**); NO_2_ (**B**); CO (**C**); and PM_2.5_ (**D**). The data is extracted from the *China Statistical Yearbook on Environment 2014* [10].

Along with the time series data and spatial distributions of the annual density of the SO_2_ (Figure 4A), NO_2_ (Figure 4B), CO (Figure 4C), and PM_2.5_ (Figure 4D) were also investigated and presented. Several key areas represented the accumulation of serious pollution, such as the Beijing-Tianjin-Hebei Region, with the annual density of the SO_2_, NO_2_, CO, and PM_2.5_ ranging from 42 μg/m^3^ to 114 μg/m^3^, from 17 μg/m^3^ to 69 μg/m^3^, from 1 μg/m^3^ to 5.9 μg/m^3^, and from 26 μg/m^3^ to 160 μg/m^3^, respectively. The maximum of the SO_2_, NO_2_, CO, and PM_2.5_ were 52 μg/m^3^, 45 μg/m^3^, 2.3 μg/m^3^, and 69 in Old Northeastern Industrial Base of China. In addition, the maximum of the SO_2_, NO_2_, CO, and PM_2.5_ were 36 μg/m^3^, 41 μg/m^3^, 2.3 μg/m^3^, and 75 in the Coastal Urban Belt.

The relationship of GDP with SO_2_ (Figure 4A), NO_2_ (Figure 4B), CO (Figure 4C), and PM_2.5_ (Figure 4D) was also explored, showing that the emissions significantly increased with the growth in GDP, whose fitting functions were *Y* = 36.46*X*^0.11^ (*R*^2^ = 0.01, *p* < 0.05), *Y* = 36.71*X*^0.15^ (*R*^2^ = 0.23, *p* < 0.01), *Y* = 2.12*X*^0.09^ (*R*^2^ = 0.01, *p* < 0.05), and *Y* = 55.07*X*^0.18^ (*R*^2^ = 0.21, *p* < 0.01). Thus, spatial pollution patterns are closely related to the distribution of social economic activities.

### 3.3. Relationship between Air Pollution and Human Health

**Figure 5:**
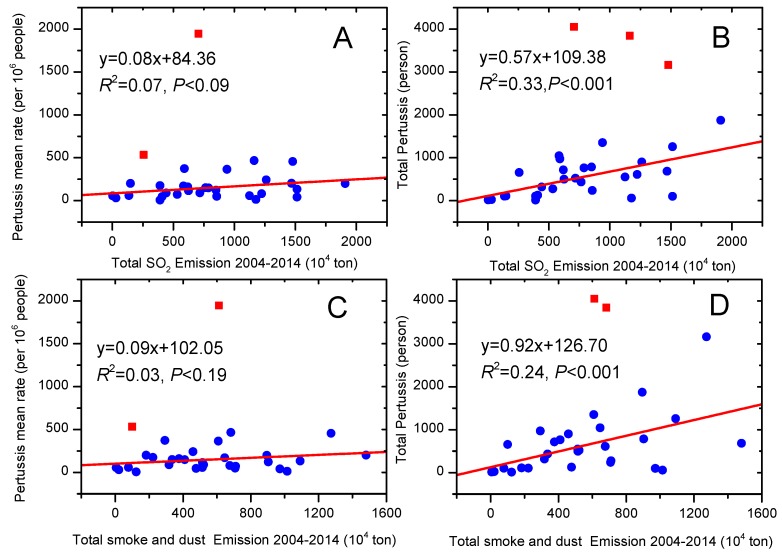
The relationships of pertussis mean rate and total pertussis, with total SO_2_ emission (**A**,**B**); total smoke and dust emission (**C**,**D**) during 2004–2014 at provincial level. Note that the red blocks are excluded from the regressions. Data resources: *China Statistical Yearbook* (2004–2015) [9] and *China Statistical Yearbook of Health and Family Planning* (2004–2015) [11].

General linear model analysis showed that there was no significant relationship between the total pertussis and total SO_2_ emission, and total smoke and dust emission (Figure 5A,C). However, the rate of total pertussis were significantly associated with total SO_2_ emissions, total smoke and dust emission during 2004–2014 (Figure 5B,D). The variation explained by both total SO_2_ emission and total smoke and dust emission were 33% (*p* < 0.001) and 24% (*p* < 0.01) for the rate of total pertussis, respectively. 

## 4. Discussion

### 4.1. The Relationship of Air Pollution with National Economic and Social Development

China is currently the world’s second largest energy producer and consumer [12]. Over the last 30 years, China has been in a blowout economic growth process, with an average annual growth rate of 8%–9%. However, environmental pollution problems (e.g., air pollution, soil acidification, water eutrophication, and potential pandemics, *etc.*) have led to an increasing focus on energy consumption and human health. Li *et al.* [13] reviewed China’s economic growth and energy consumption, and suggested promoting a low carbon economy. Meanwhile, Zhang *et al.* [14] evaluated the relationship among economic growth, energy consumption, emissions, and environmental protection investments in China. Li *et al.* [15] also analyzed the energy consumption-economic growth relationship and carbon dioxide emissions in China, and determined the existence of a positive long-run co-integrated relationship between real GDP *per capita* and energy consumption variables, and hold that a 1% increase in the PGDP increases the consumption of energy by approximately0.48%–0.50% and accordingly increases the carbon dioxide emissions by about 0.41%–0.43% in China. In this study, the same trends of PGDB, coal consumption, and the demand for steel were observed (Figure 1), with the similar tipping points in 2001, 2002, and 2004, respectively.

A number of studies have been undertaken to determine the relationship between economic development, energy consumption, and air pollution [16,17]. Our study also documented that economic development has a significant influence on the annual changes in coal consumption (*R*^2^ = 0.99, *p* < 0.001), annual steel production (*R*^2^ = 0.99, *p* < 0.001), and SO_2_ emission (*R*^2^ = 0.98, *p* < 0.001, Figure 2). China’s greenhouse gas emissions are likely to peak, and then begin to taper off around 2025 approximately [18], which has been confirmed in the latest Paris COP 21 conference with subsequent adjustment to the energy structure and other activities. This means that China’s greenhouse gas emissions could begin to decline within 10 years, five years earlier than expected, being beneficial to climate change alleviation and environmental protection. The shift was partly from a massive commitment to renewable clean energy, which still has great potential. China has been planning to initially cap emissions from six industrial sectors, *i.e.*, power generation, metallurgical, nonferrous metal, building materials, chemicals, and aviation. Several technical innovations have been launched to monitor, predict, and act to overcome and reduce air pollution. China has been the top investor in wind and solar power worldwide. It has also begun replacing old coal plants with cleaner and newer stations. For instance, water spray geoengineering has been used to mitigate haze in China’s cities [19], and a luminance reference model was used to monitor haze by image interpretation [20,21]. Furthermore, an odd-even day vehicle prohibition rule has occasionally been implemented during big events, e.g., APEC (Asia-Pacific Economic Cooperation) or the Olympic Games have even been announced during regional holidays. If the Chinese economy continues to grow at a high rate, GHG emissions will ensue, and Chinese leaders will continue to face the dilemma to make a tradeoff between economic growth and emissions [13]. China is still attempting to determine an equilibrium between environmental protection and economic development.

### 4.2. The Relationships between Human Health and Air Pollution

The distribution of population density appears consistent by visual inspection with the spatial pattern of pollution and social economic activities (Figure 3 and Figure 4). The results suggest that human activities have impacts on the environment from different aspects, and reminds us that a large portion of the population has been significantly exposed to polluted areas. As compared to the negative impacts on adults, children have been known to suffer more showing increased rate of respiratory symptoms, decreased lung function, and increased incidences of chronic cough, bronchitis, and conjunctivitis with long-term exposure [22]. Indoor air pollution from household biomass fuels showed a strong and significant risks remained in risk for exposed young children than those living in households using cleaner fuels or being otherwise less exposed [22]. A case study of air monitoring for particulate matter <2.5 μm in diameter (PM_2.5_) and polycyclic aromatic hydrocarbons (PAHs) indicated that the strongest effects existed between air pollution linked with morbidity and mortality on the very young and the elderly. Preschool-age children may be particularly vulnerable to air pollution–induced illnesses [23]. It has important public health implications for the present day and the future to better understand the impacts of the result of air pollution. If the government fails to find a balance between economic growth and environmental protection in a timely manner, the vast majority of people will face serious health risks from environmental deterioration.

Recently, haze pollution has attracted much attention for its significant effect on public health [24]. Previous research reported that air pollution might have a great impact on pulmonary health [25,26], and particularly the number of paediatric patients with pneumonia dramatically increased in China [27]. Fine particulate matter (PM_2.5_) has been highlighted recently for its adverse effect on health, as it can easily bypass normal body defense mechanism and penetrate deep into the alveoli of the lungs. One case study presented the impact of haze to lung health from the forest fires in Singapore, whose supplementary findings by scanning of the haze particles showed that 94% of the particles in the haze were PM_2.5_. Besides, it also caused an increased accident and emergency attendance for haze-related conditions though no significant increase in hospital admissions or in mortality [28]. In Shanghai, China a case study of health risk assessment was conducted to compare between haze and non-haze periods [24]. In several major cities, over a quarter of a million people may face premature death due to high levels of air pollution. Our findings suggest that both the pulmonary tuberculosis and the pertussis were closely related to total smoke and dust emissions and total SO_2_ emissions (Figure 5). Spatially, higher air pollution was observed in Northern and Southeastern part of China (Figure 4), and the same results were found via MODIS (Moderate Resolution Imaging Spectroradiometer) remote production [29]. As a matter of fact, health impacts and health-related economic losses were quite large [30]. The haze in January 2013 might lead to 253.8 million US$ lost, accounting for 0.08% of the total 2013 annual gross domestic product (GDP) of Beijing. In a word, haze has negative impacts on public health [26], tourism [31], education [26,32], traffic [33], economic development [34] and so on.

### 4.3. Limitations of the Current Study

In the present study, the relationship between air pollution, economic development, and human health was explored at temporal and spatial scales. Although significant relationship among these variables was observed, part of the uncertainty comes from the data on air pollution. The data on SO_2_, NO_2_, CO, and PM_2.5_ were extracted from the *China Statistical Yearbook on Environment 2014*, in other words, we explored the relationships of GDP with SO_2_, NO_2_, CO, and PM_2.5_ using the statistics data in 2013. The lack of long term data on air pollution could have led to the above conclusions, which had recommended that long-term effects and morbidity in China is still not adequate, in particular, evidence of constituent-associated health effects, though short exposures to PM_10_ and PM_2.5_ are associated with increases in mortality [35]. Thus, we need to collect detailed information to define those risks of human health and air pollutions in future.

In addition, it has been referred that the total energy consumption had been understated by 10% from 2002 to 2012 [36], so that emissions of soot and PM_2.5_ might be also understated to a certain extent, which are coupling well with total energy consumption in the time series according to our analysis and results, so is the carbon intensity (*China City Statistical Yearbook 2013*). Nevertheless, there are quite a few actual measurements representative of the mixed fuels in China [36], and the carbon intensity and energy consumption are conflicting with uncertain sources and factors, resulting in notably lower emissions from coal and cement production factors than the recommended default values, or other previous estimations [2,36,37]. Our findings highlight that tipping point of all indexes appeared in 2001–2004 (Figure 1 and Table 1). In accordance to the previous studies, they have taken period from 2002 till recent years into account for further mining. However, uncertainties also exist from various sources, in particular spatial distribution patterns and multiple factors. It should be noted that China has drawn attention to domestic actions to combat the challenge as promised at the Paris conference to deepen practical action in the areas of clean energy technologies, energy conservation and efficiency, renewable energy, sustainable transportation, low-carbon urbanization and adaptation.

### 4.4. The Long-Term National Strategic Development Plans

How can air pollution be reduced? A series of important policy suggestions had been proposed previously. Fang demonstrated that market openness, institutional innovation, and policy integration are important to improve the sustainable development of renewable energy [38]. Wang and Liu suggested developing new techniques to control the emission of pollutants and replacing fossil fuels with clean fuel [6]. Fluctuation of energy intensity is mainly due to technology advances and the corresponding changes in industrial structure [39]. How to decrease energy intensity? Chinese government must cut down the proportion of coal in energy consumption, increase the utilization efficiency of coal and promote the up-gradation of the economic structure. Furthermore, analysis of factors that may relate to energy intensity should be fully conducted before making energy policies [18]. The energy intensity has started to show slight decline since 2006, attributed to the government efforts [40]. It can relieve the pressure on the central heating system in northern China by regulating the quality of gasoline and coal, gradually replacing coal with cleaner fuel, encouraging consumers to purchase higher energy efficiency vehicles or new energy automobiles, and constructing sustainable buildings.

**Figure 6:**
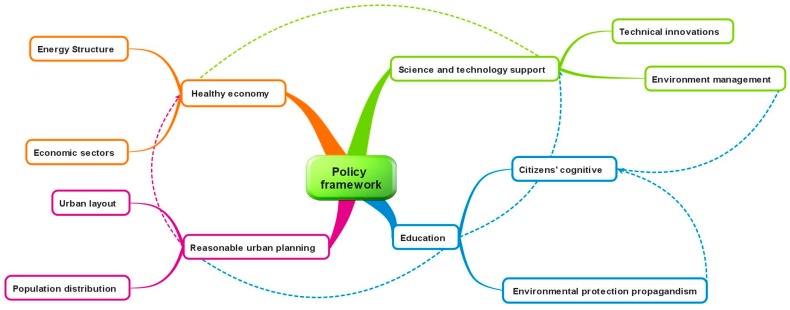
The new mind map of the policy framework.

The ubiquitous exposure as air pollution has important public health implications. It is reasonable to quantify the risk more precisely, to determine the degree of reduction in exposure required to significantly improve health, and to establish the effectiveness of interventions [22]. From our perspective, unreasonable industry and energy structure has significant negative impacts on energy consumption and environmental quality. Meanwhile, scientific urban layout and reasonable population distribution also act as underlying factors. Technical innovations are the key approaches promoting healthy economic development. Environmental management, education, and citizen’s continuous learning are guarantees for long-term development of the Chinese economy. A reasonable balance must be found to achieve a healthy economy while respecting the natural environment, so that, being a precondition, to establish policy and laws have to be of the utmost importance (Figure 6).

## 5. Conclusions

Economic development and energy consumption has increased dramatically in China over the past decades. In general, the most polluted areas appear in relatively concentrated and developed economic regions. To achieve the goal of eliminating haze and pursuing an environmentally friendly and sustainable economic development, energy and industrial structures must be adjusted and optimized, and energy efficiency must be improved as soon as possible. In light of limited available information, additional studies at utilizing a variety of technologies and innovative energy are recommended. In future, China should attach more importance not only to technical progress but optimizing its sector structure, and lowering its investment ratio to save more energy. Unquestionably, implementing effective environmental management policies, establishing suitable energy policies, and improving understanding and environmental awareness of citizens are also very important measures [26,27,30]. Whether the trend in rising energy intensity continues will be significant for China and the rest of the world.

## Figures and Tables

**Table 1:** The statistical descriptions of annual coal consumption, the demand for steel, the total SO_2_ emission, and the economic growth trend.

Items	Function 1	Tipping Point	Function 2	*R*^2^	*p*
Annual coal consumption	*Y* = 0.02*X* − 37.47	2002	*Y* = 0.22*X* − 434.83	0.99	*p* < 0.001
The demand for steel	*Y* = 4.99*X* − 9849.96	2001	*Y* = 60.36*X* – 120,650.98	0.99	*p* < 0.001
Total SO_2_ emission	*Y* = 1.03*X* − 2022.61	2002	*Y* = 3.77*X* + 5405.29	0.99	*p* < 0.001
Economic growth (PGDP)	*Y* = 5672.81*X* − 1.13 × 10^7^	2004	*Y* = 46,911.08*X* − 9.36 × 10^7^	0.99	*p* < 0.001
